# PVBase: A MALDI-TOF MS Database for Fast Identification and Characterization of Potentially Pathogenic *Vibrio* Species From Multiple Regions of China

**DOI:** 10.3389/fmicb.2022.872825

**Published:** 2022-05-17

**Authors:** Tingting Liu, Lin Kang, Jinglin Xu, Jing Wang, Shan Gao, Yanwei Li, Jiaxin Li, Yuan Yuan, Bing Yuan, Jinglin Wang, Baohua Zhao, Wenwen Xin

**Affiliations:** ^1^Hebei Key Laboratory of Animal Physiology, Biochemistry and Molecular Biology, College of Life Sciences, Hebei Normal University, Shijiazhuang, China; ^2^State Key Laboratory of Pathogen and Biosecurity, Beijing Institute of Microbiology and Epidemiology, Academy of Military Medical Sciences (AMMS), Beijing, China

**Keywords:** *Vibrio* species, database, pathogenic, clinical strain, environmental strain, regional strain

## Abstract

The potentially pathogenic species of the genus *Vibrio* pose a threat to both humans and animals, creating medical burdens and economic losses to the mariculture industry. Improvements in surveillance and diagnosis are needed to successfully manage vibriosis outbreaks. Matrix assisted laser desorption/ionization time-of-flight mass spectrometry (MALDI-TOF MS) can provide rapid diagnosis and has been widely used in the identification of *Vibrio* spp. The main weakness of this technology is the limited number of strains and species of *Vibrio* in the existing commercial database. Here, we develop a new in-house database named PVBase containing 790 main spectra projections (MSP) of ten *Vibrio* species that come from various regions of China and include abundant clinical and environmental strains. PVBase was validated through a blind test of 65 *Vibrio* strains. The identification accuracy and scoring of *Vibrio* strains was greatly improved through the addition of PVBase. Identification accuracy increased from 73.4 to 100%. The number of strains with identification scores above 2.2 increased from 53.1% to 96.9% and 53.1% of strains had an identification score above 2.59. Moreover, perfect discrimination was obtained when using all of the MSPs created for the *Vibrio* species, even for very closely related species such as *V. cholerae*, *V. albensis*, and *V. mimicus* or *V. alginolyticus*, *V. parahaemolyticus*, and *V. harveyi*. In addition, we used phyloproteomic analysis to study whether there are differences in protein fingerprints of different regions or pathogenic strains. We found that MSP characteristics of *Vibrio* species were not related to their region or source. With the construction of PVBase, the identification efficiency of potentially pathogenic *Vibrio* species has been greatly improved, which is an important advance for epidemic prevention and control, and aquaculture disease detection.

## Introduction

*Vibrio* is a Gram-negative bacterium that can have short rod, curve, S or spiral shapes and is widely distributed in sea water and in marine animals in estuaries, bays, and coastal waters. As of 10 December 2021, a total of 133 *Vibrio* species had been identified^1^. While not all *Vibrio* species are pathogenic, about 20 pathogenic *Vibrio* species are recognized worldwide and about 12 of these can cause disease in humans (Baker-Austin et al., 2018). Sixteen species are known to cause pathogenesis in aquatic organisms (Nag et al., 2020).

Some species of *Vibrio* spp. are well-known to be related to human gastrointestinal diseases, wound infections and sepsis, causing many human diseases. The most common pathogenic *Vibrio* are *V. parahaemolyticus*, *V. cholerae* and *V. vulnificus*. The associated disease, cholera, is an international quarantine infectious disease (Conner et al., 2016). An estimated 35 million people worldwide are infected with cholera, and approximately 140 thousand people die from it every year (Ali et al., 2015). In the last two centuries there have been seven cholera pandemics and numerous outbreaks, posing a major public health threat to 175 countries in Asia, Africa and the Americas (Banerjee et al., 2014). *V. parahaemolyticus* infections are related to eating raw or undercooked contaminated seafood, and wounds exposed to contaminated water (Li et al., 2019). *V. parahaemolyticus* is one of the leading causes of foodborne diseases in China and America (Bonnin-Jusserand et al., 2019). *V. vulnificus* is a conditional pathogen and generates sporadic infections, and is often fatal and almost all cases occur in people with underlying diseases. *V. vulnificus* is classified as the pathogen of Class 3 infectious diseases in China (Vezzulli et al., 2016). Some species of *Vibrio* are pathogenic bacteria that affect aquatic animals and can cause large-scale aquaculture infections and economic losses in the aquaculture industry (de Souza Valente and Wan, 2021). For example, *V. harveyi*, *V. alginolyticus*, *V. rotiferianus* and *V. anguillarum* are considered “source of disaster” in the marine fish and shellfish aquaculture industry (Frans et al., 2011). Bacterial culture of the hepatopancreas tissues of moderately and severely diseased *Penaeus vannamei* cultured in clear sea water confirmed *V. harveyi* and *V. rotiferianus* (Aguilera-Rivera et al., 2019). For pathogen transmission control and farm handling, veterinarians and researchers need reliable bacterial identification tools.

Some *Vibrio* species have high genome homology, such as *V. alginolyticus* and *V. parahaemolyticus* (Salamone et al., 2019) or *V. cholerae* and *V. mimicus* (Wang et al., 2011). This makes the characterization, classification, and identification of *Vibrio* species particularly problematic, especially for the closely related species (Moussa et al., 2021). It is difficult to accurately distinguish among bacterial species using 16S rRNA sequencing (Johnson et al., 2019). Whole genome sequencing is considered the gold standard for identifying *Vibrio*, but it is expensive and time-consuming (Ranjan et al., 2016). Quantitative real-time PCR is already used for *Vibrio* identification, however, it requires specific primers and probes for each pathogen (Li et al., 2016). In recent years, matrix assisted laser desorption/ionization time-of-flight mass spectrometry (MALDI-TOF MS) has been widely used in the identification of clinical pathogenic microorganisms (Swiner et al., 2020) related to food (Quero et al., 2019) and environmental safety (Santos et al., 2016). Compared with traditional phenotyping techniques or molecular biology, MALDI-TOF MS is a fast, accurate and economical identification method that can analyze the entire microorganism with a small amount of sample preparation and a greatly reduced identification time (30 min). MALDI-TOF MS can also identify microorganisms that are difficult to culture, such as microaerobion, anaerobes, mycobacteria and fungi, leading to become a recognized milestone in the rapid identification of microorganisms (Biswas and Rolain, 2013; Swiner et al., 2020). Previous studies have demonstrated that MALDI-TOF MS is a valuable tool for discriminating among closely related species (Schaumann et al., 2018) and *Vibrio* spp. are no exception (Moussa et al., 2021). Although scholars have constructed databases related to *Vibrio* spp. and achieved outstanding results, they also pointed out that due to the intra- and inter-species coefficient of variation, a sufficient number of main spectra in the database is crucial for reliable and safe species identification (Erler et al., 2015; Moussa et al., 2021).

At present, the latest database of MALDI BioTyper Library ver. 11.0.0.0 (Bruker MBT Library, modified on 11th July 2021) contains 54 species of *Vibrio*. The following species (number of strains) have more than five strains in the database: *V. vulnificus* (11), *V. parahaemolyticus* (9), *V. harveyi* (7), and *V. diazotrophicus* (6). All other *Vibrio* species in the database are represented by fewer than five strains, and most have only one strain. This limited number of strains can greatly affect the identification accuracy of potentially pathogenic *Vibrio* species. In this study, we used 790 strains of *Vibrio* spp., collected from various regions of China and purchased from the standard bacterial library, to construct an in-house database of potential pathogenic *Vibrio* spp. that we call PVBase. PVBase increased the diversity of known strains in China and improved accuracy of the identification of potential pathogenic *Vibrio* spp. in different application scenarios by MALDI-TOF MS. To explore the genetic relationship and the characteristics of strains of *Vibrio* species from different regions, as well as clinical vs. environmental strains, we performed dendrogram analysis of different *Vibrio* species from the Bruker MBT Library and PVBase. In addition, we validated the newly created database through a blind test using 65 strains (absorbed into the database after the blind test was completed.). The establishment of PVBase enables accurate, rapid identification of a large number of *Vibrio* spp. and contributes to enhancing clinical diagnosis and treatment, restriction of pathogen transmission, and control of large-scale spread of diseases.

## Materials and Methods

### Bacterial Strains

**FIGURE 1:**
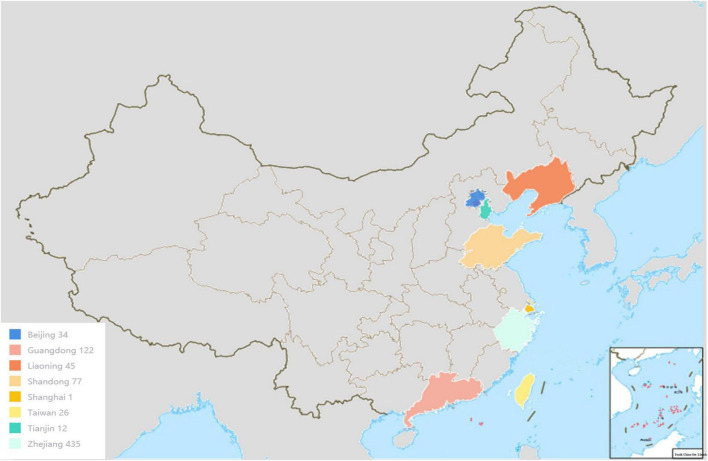
The main geographical distribution of *Vibrio* strains.

A total of 790 potentially pathogenic *Vibrio* strains were analyzed. Briefly, most the strains were obtained from Zhejiang, Guangzhou, Guangdong, Liaoning, Taiwan, Shandong, Tianjin, Shanghai, and Beijing, and a small number of strains were shared from the Marine Culture Collection of China (MCCC) and the American Type Culture Collection (ATCC). The geographic information of the strains was shown in Figure 1. In addition, the strains included 110 clinical strains and 259 environmental strains. There were 65 strains out of 790 strains used for blind testing. Detailed information on strains is provided in Supplementary Table 1. All strains had been previously well-characterized by whole genome sequencing.

### Bacteria Culture Conditions

Before all experiments, strains were grown on marine agar 2216 medium (Becton Dickenson, Franklin Lakes, NJ, United States), and incubated for 24–48 h, at their appropriate growth temperature. After this, a single colony forming unit (CFU) was plated and incubated for 24 h, at growth temperature, to ensure purity of the isolates. The growth temperature of *V. alginolyticus*, *V. harveyi*, *V. fluvialis*, *V. fischeri*, *V. navarrensis*, *V. metschnikovii*, and *V. vulnificus* was 30°C and for *V. cholerae*, *V. parahaemolyticus* and *V. mimicus* growth temperature was 37°C. Type strains were incubated at the recommended temperature.

### Sample Preparation

For database entry, strains were prepared with the formic acid extraction method to ensure a high quality of spectra. Fresh colonies were picked with a 1 μL inoculation loop and placed in 300 μL of LC–MS water (Thermo Fisher, Waltham, MA, United States). A volume of 900 μL pure ethanol (Thermo Fisher) was added to sterilize the bacteria and denature proteins. The tube was vortexed for 1 min and centrifuged at 13,000 × *g* for 2 min. The supernatant was discarded and the pellet was centrifuged a second time to remove ethanol residues and then dried at room temperature. Pellets were dissolved into 30 μL each of 70% formic acid (Thermo Fisher) and acetonitrile (Thermo Fisher). The solution was carefully mixed by pipetting up and down and then centrifuged at 13,000 × *g* for 2 min. Subsequently, 1 μL of supernatant was spotted onto an MSP 96 target polished steel BC plates in eight replicates and overlaid with 1 μL HCCA (α-Cyano-4-hydroxycinnamic acid; Bruker, Germany) matrix solution (HCCA dissolved in 50% acetonitrile, 47.5% LC–MS water and 2.5% trifluoroacetic acid) after air-drying. All *V. cholerae* from Zhejiang were processed in the Zhejiang Centers for Disease Control laboratory according to the above-mentioned treatment methods and carried to our laboratory for the collection of the spectra.

### Acquisition of MALDI-TOF MS Spectra

Following the manufacturer’s directions, a bacterial test standard (BTS) was used on each acquisition plate. A BTS can calibrate the instrument before each acquisition session to ensure quality of the acquisition.

Spectra were acquired using the Microflex LT system (Bruker). Each spot was measured three times, resulting in 24 single spectra for each strain. The software flexControl v. 3.4 automatically acquired the spectrum of each spot through AutoXecute. Signal intensity of the highest peak in the spectra was maintained between 1,000 and 5,000 by fine-tuning the laser intensity. The linear positive ion mode was used as the acquisition mode of the instrument and parameters were set as follows: lens, 8.5 kV; ionsource1, 20 kV; ionsource2, 18.1 kV; mass range: 2,000–20,000 Da; laser frequency: 60 Hz; Shots/Spectrum: 240. The instrument was calibrated using calibrators (4000–10,000 Da) with molecular weights of 3,637.8 Da, 5,096.8 Da, 5,381.4 Da, 6,255.4 Da, 7,274.5 Da, 10,300.1 Da, 13,683.2 Da, and 16,952.3 Da. The masses of the measured spectrum were within the tolerance range of ± 300 ppm from nominal value.

### Database Construction

Baseline subtraction and smoothing were performed after importing the spectra into flexAnalysis v. 3.4 (Bruker). Next, we searched the spectra set for flatline spectra, sweet spot outliers and anomalies, removing such spectra by closing out of the set. A minimum of 18 spectra were required for main spectra projections (MSP) creation; where necessary the measurement was repeated using a new sample preparation to achieve this. In the peak shift of individual masses, we selected peaks from 3,000 to 10,000 Da in steps of 1,000. The allowed peak shift between the spectra with the smallest and the largest mass was 500 ppm. Finally, we selected the remaining spectra in flexAnalysis v. 3.4 and used these data to create the MSP through MALDI BioTyper Compass Explorer v. 4.1 software. All MSPs were registered in our in-house database, PVBase. In addition, we established separate databases of different sizes for *V. parahaemolyticus*, *V. cholerae*, *V. alginolyticus*, *V. harveyi*, and *V. vulnificus* to observe the changes in the highest matching scores identified by different sizes of MSP present.

### MALDI-TOF MS PVBase Validation

790 strains of *Vibrio* spp. were involved in this study, 65 strains used in the blind test and 725 strains used in the PVBase database. The 65 strains for blind test were first identified using Bruker MBT Library, and then identified using a database merged Bruker MBT Library with the PVBase. Then we compared the identification results of the two databases, and analyzed the changes in the identification accuracy and accuracy scores of related strains. We also used the Moussa database (Moussa et al., 2021) to identify the blindly tested strains, and related results are presented in Supplementary Table 2.

### Phyloproteomic Analysis

Dendrogram analyses were performed using MALDI BioTyper Compass Explorer v. 4.1 software. The distance measurement was set to “correlation” and the linkage algorithm was set to “average.” Principal components analysis (PCA) and composite correlation index (CCI) were used to analyze the differences between and within the species of *Vibrio* through ClinPro Tools 3.0. The dendrogram obtained from the PCA analysis indicates how close spectra are to one another. The default parameter for the PCA dendrogram analysis was “hierarchical,” the distance measurement was “correlation,” and the link algorithm was “average.” In general, it is considered statistically different when the sum of the variances of PC1 and PC2 is greater than 80%. All measured spectra were loaded and the CCI was calculated using MALDI BioTyper Compass Explorer 4.1. Parameters were set as follows: mass lower bound, 3000; mass upper bound, 12000; resolution, 4; and CCI parameter interval, 8. The CCI value is between 0 and 1, where 0 indicates spectra are completely uncorrelated and 1 indicates spectra are completely correlated.

## Results

### PVBase Establishment

In this study, a total of 790 strains were collected and included in the database. In order to select the most representative spectra and merge them into strain-specific MSP, we searched 790 spectra set for sweet spot and removed anomalies by closing out of the set by flexAnalysis ver.3.4 (Bruker, Germany). All 790 MSPs were registered in an in-house database called PVBase. The numbers of MSP for the *Vibrio* species that are potentially pathogenic in the Bruker MBT Library and our PVBase are shown in Table 1. *V. vulnificus*, *V. cholerae* and *V. parahaemolyticus* had a wide range of sources and relatively large numbers, while the remaining *Vibrio* species had relatively small number of strains.

**TABLE 1:** Numbers of main spectra projections (MSP) for the *Vibrio* species that are potentially pathogenic in the Bruker MBT Library and in our PVBase.

Species	Bruker MBT Library MSP number	PVBase MSP number
*V. alginolyticus*	5	52
*V. harveyi*	7	20
*V. navarrensis*	5	9
*V. vulnificus*	11	121
*V. mimicus*	1	3
*V. metschnikovii*	2	3
*V. cholerae*	0	197
*V. fluvialis*	3	11
*V. furnissii*	3	4
*V. parahaemolyticus*	9	370

### The Effect of Database Size on Identification Results

**FIGURE 2:**
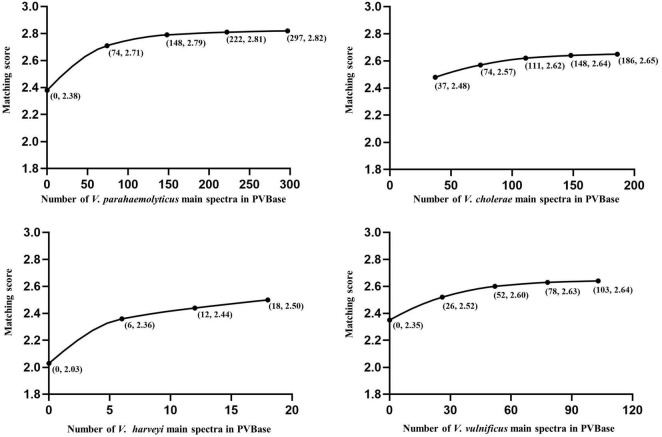
Correlations between the number of main spectra filed in PVBase and expected mean of highest matching scores. Data for *V. parahaemolyticus*, *V. cholerae*, and *V. vulnificus* are provided as examples. Numbers in parentheses below points represent the number of strains added in a single addition and the matching score.

**FIGURE 3:**
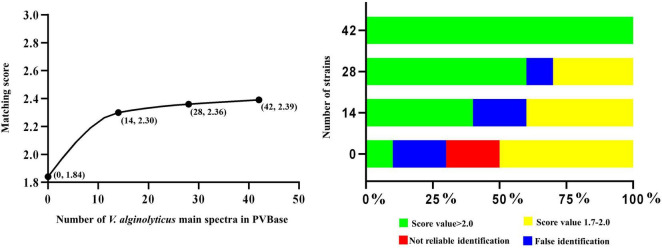
Correlation between number of main spectra for *V. alginolyticus* archived in the PVBase and the highest expected matching score or identification accuracy.

We looked for an effect of database size on identification by establishing separate databases of different sizes of *V. parahaemolyticus*, *V. cholerae*, *V. alginolyticus*, *V. harveyi*, and *V. vulnificus* to observe changes in the highest matching scores with number of MSP present. Except for *V. cholerae*, which is not contained in Bruker MBT Library (Table 1), the initial scores of *V. parahaemolyticus*, *V. alginolyticus*, *V. harveyi*, and *V. vulnificus* were all obtained by matching the Bruker MBT Library. In *V. harveyi*, the highest matching score had been on an upward trend, and had not yet reached saturation as the MSP increases. It further illustrated the necessity of increasing the number of strains in the database. For *V. parahaemolyticus*, *V. vulnificus*, *V. alginolyticus*, and *V. cholerae* the highest matching score also increased initially, but then plateaued, suggesting saturation was reached (Figures 2, 3). The results support the hypothesis that a sufficient number of main spectra in the database is crucial for highly probable MALDI-TOF MS species classification.

The importance of increasing database size is further clarified through in-depth consideration of results with *V. alginolyticus*. The initial matching score of *V. alginolyticus* obtained by matching the Bruker MBT Library was 1.65–1.85, and the best matches for strains were *V. mytili*, *V. parahaemolyticus* or *V. harveyi*, followed by *V. alginolyticus*. In addition, some strains also had not been accurately identified. With the continuous increased in the number of MSPs in PVBase, the identification accuracy of *V. alginolyticus* had been significantly improved. When the number of MSPs was 30, all *V. alginolyticus* were accurately identified, and the identification score increased to approximately 2.4 (Figure 3).

### MALDI-TOF MS PVBase Validation Through a Blind Test

**FIGURE 4:**
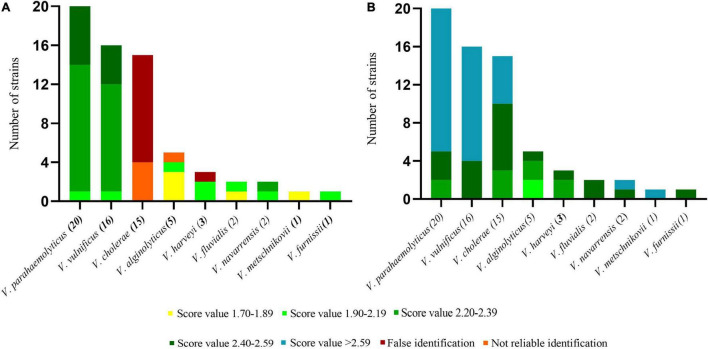
Blind test of identification of *Vibrio spp.* using MALDI-TOF MS. **(A)** Results based on the Bruker MBT Library. **(B)** Results based on the Bruker MBT Library and PVBase combined.

Of the 790 strains collected in this study, 65 strains were used for the blind test, and the remaining strains were used as databases for verification The test spectra were first identified using the Bruker MBT Library (Figure 4A). Four strains of *V. cholerae* were not identified, and the remaining 11 strains were all identified as *V. albensis*. In addition, one strain of *V. harveyi* was erroneously identified as *V. parahaemolyticus*. The remaining *Vibrio* species were correctly identified, and the initial identification scores of *V. parahaemolyticus*, *V. vulnificus* and *V. navarrensis* were highest. The test strain identifications were then obtained through PVBase and the Bruker MBT Library together (Figure 4B). After the PVBase database was added, all *Vibrio* species were correctly identified, and the identification score was generally greater than 2.2; identification scores of some strains of *V. parahaemolyticus*, *V. vulnificus*, *V. cholerae*, *V. navarrensis* and *V. metschnikovii* exceeded 2.59. Thus, the addition of the PVBase database greatly improved the accuracy and matching scores when identifying *Vibrio* species.

### Phyloproteomic Analysis of *Vibrio* Species

**FIGURE 5:**
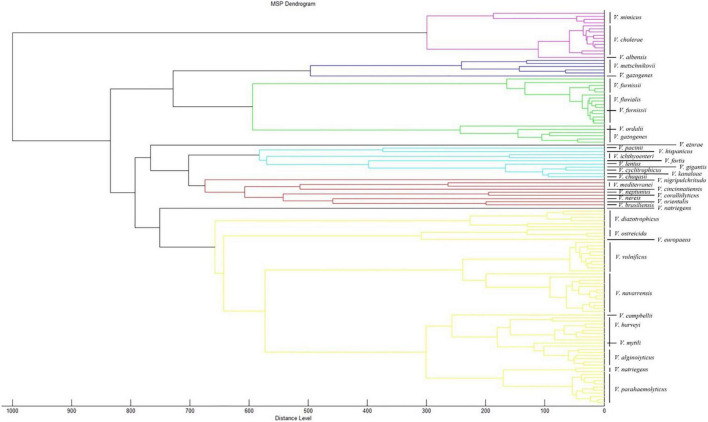
Dendrogram analysis of different *Vibrio* species. 77 strains of all *Vibrio* species were randomly selected from PVBase and 48 strains of all *Vibrio* species were randomly selected from Bruker MBT library. The dendrogram analysis was performed using MALDI BioTyper Compass Explorer v. 4.1.

To observe the genetic relationship between *Vibrio* spp., we randomly selected 77 strains of all *Vibrio* species, which included those in PVBase and 48 strains of all *Vibrio* species included in the Bruker MBT Library as representatives. We then performed a dendrogram analysis using MALDI BioTyper Compass Explorer ver.4.1 software (Figure 5). Accurate distinction was feasible for most species of *Vibrio* including *V. mimicus*, *V. cholerae* and *V. albensis*, which were previously reported indistinguishable species (Manjusha et al., 2013). Only a few *Vibrio* species could not be clearly distinguished. The MSP of one strain of *V. furnissii* clustered in the group of *V. fluvialis*. In addition, the similarity between *V. ordalii* and *V. gazogenes* or *V. mytili* and *V. harveyi* was also high, and these species pairs cannot be distinguished easily by MSPs. However, we had few strains of these indistinguishable *Vibrio* species, so the relevant conclusions need to be further verified.

**FIGURE 6:**
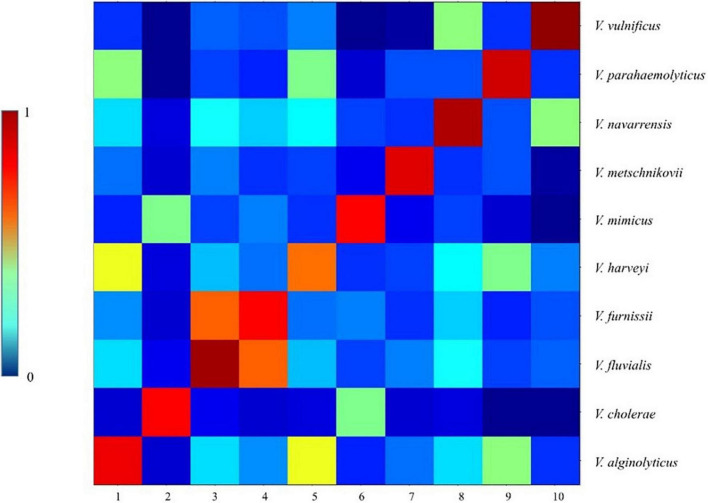
CCI matrix of different *Vibrio* species. 77 strains of all *Vibrio* species were randomly selected from PVBase, which were consistent with the 77 strains selected for dendrogram analysis. A CCI analysis was performed using ClinPro Tools 3.0.

We performed a CCI analysis on the 77 strains of *Vibrio* species randomly selected from PVBase (Figure 6). Consistent with dendrogram analysis, the CCI matrix showed that *V. fluvialis* and *V. furnissii* were relatively difficult to distinguish because of their high similarity. In addition, similarity between the *Vibrio* species obtained by the color depth of the CCI matrix was consistent with the dendrogram analysis.

### Analysis on the Characteristics of *Vibrio* Species Found in Different Regions

**FIGURE 7:**
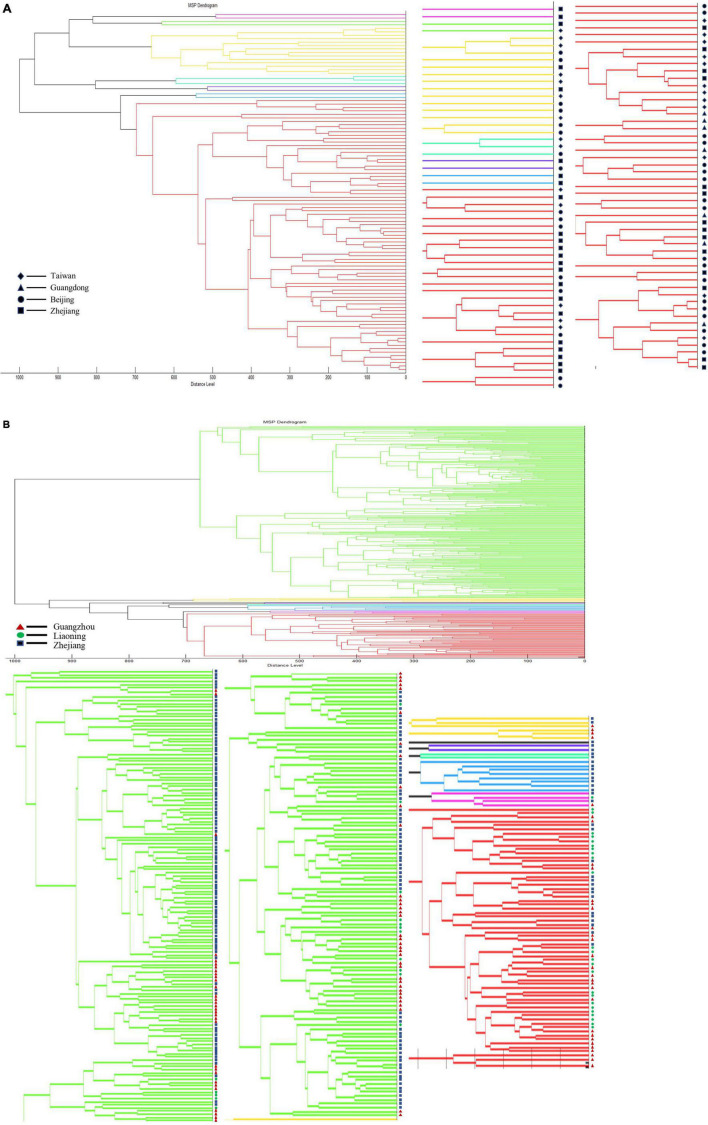
Dendrogram analysis of strains in different regions. **(A)** Dendrogram analysis of *V. vulnificus* in different regions. **(B)** Dendrogram analysis of *V. parahaemolyticus* in different regions.

To explore whether *Vibrio* species have regional characteristics, we ran separate cluster analyses on strains of *V. vulnificus* and *V. parahaemolyticus*. *V. vulnificus* was collected from Taiwan, Beijing, Guangdong, and Zhejiang. Dendrogram analysis showed that using a distance level of 900, the species could be divided into three groups, but there was no obvious correlation between MSP characteristics of the strain and region (Figure 7A). The analysis of *V. parahaemolyticus* reached a similar conclusion. Dendrogram analysis on *V. parahaemolyticus* collected from Zhejiang, Liaoning and Guangzhou indicated that *V. parahaemolyticus* could be divided into two groups using a distance level of 950, but that group characteristics were not directly related to region (Figure 7B). In addition, dendrogram analysis showed that the distribution of strains located in Liaoning in northern China differed to some extent from those of Zhejiang and Guangzhou located in southern China. Therefore, we performed PCA analysis on *V. parahaemolyticus* according to the division of north and south regions, but no statistical difference was obtained (Supplementary Figure 1). The sum of the variances of PC1 and PC2 was not reached 80%.

### Analysis on the Characteristics of Clinical Versus Environmental Strains of *Vibrio* Species

**FIGURE 8:**
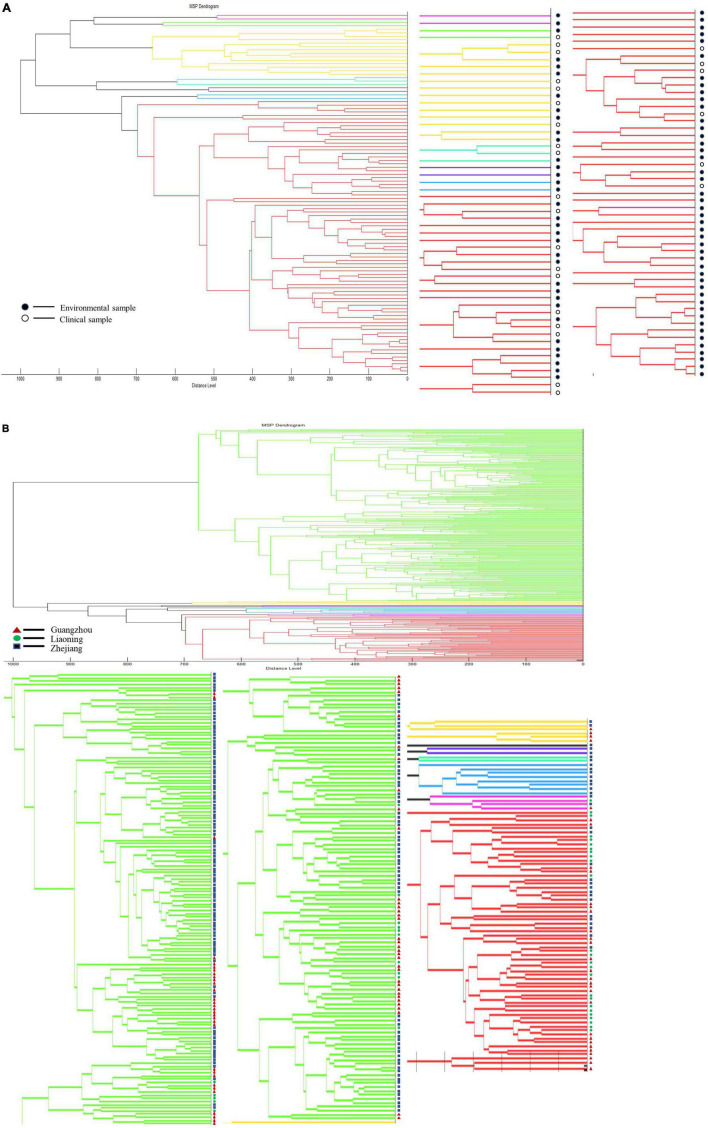
Dendrogram analysis of clinical strains and environmental strains. **(A)** Dendrogram analysis of *V. vulnificus*. **(B)** Dendrogram analysis of *V. parahaemolyticus*.

We clustered *V. vulnificus* and *V. parahaemolyticus* to explore whether either species had characteristic differences between clinical and environmental strains. Dendrogram analysis for *V. vulnificus* with a distance level of 900, divided the species into three groups, but there is no obvious correlation with clinical vs. environmental strain type (Figure 8A). Likewise, *V. parahaemolyticus* could be divided into two groups using a distance level of 950, but the groupings were not related to strain type (Figure 8B).

## Discussion

Although isolated strains are necessary for MALDI-TOF MS identification, which is time consuming, it plays a pivotal role in the identification of *Vibrio* spp., providing high sensitivity and high throughput. MALDI-TOF MS can detect the activity of bacteria, and also has obvious advantages in large-scale detection in clinical and farm handling. Database size is a key factor affecting the accuracy of MALDI-TOF MS identification. We constructed an in-house database of 790 strains of *Vibrio* spp. that greatly supplemented the Bruker MBT Library with potentially pathogenic *Vibrio* species. Increasing the number of strains in the database improved the accuracy of identification, and increasing the number of *Vibrio* species expanded the scope of identification (Mougin et al., 2020). We took *V. parahaemolyticus*, *V. cholerae*, *V. alginolyticus*, *V. harveyi*, and *V. vulnificus*, each of which have a large number of strains, as examples with which to explore the impact of increasing the number of strains in the database. With the large number of *V. parahaemolyticus*, *V. cholerae*, and *V. vulnificus*, the matching score gradually rose with increased number of MSPs until it tended to saturate. These values where matching score saturates represent the minimum number of main spectra required to account for intraspecific variation. Although the total number of added strains of *V. alginolyticus* was only 30, the identification score also appeared to become saturated. This may be due to low intra-species variability in *V. alginolyticus*, so that relatively few strains were required to saturate the identification score. After the establishment of PVBase, the blind test experiment showed strong improvement of identification accuracy and score for *Vibrio* species with the database expansion. Identification accuracy of the 65 strains in the blind test increased from 73.4 to 100%. The percentage of strains with identification scores above 2.2 increased from 53.1% to 96.9%. In addition, 53.1% of strains had identification scores above 2.59.

The dendrogram analysis of *Vibrio* species in the Bruker MBT Library and PVBase combined revealed good differentiation at the species level for *Vibrio*. *V. cholerae*, *V. mimicus* and *V. albensis* are closely related, as previously observed (Wang et al., 2011), but they are completely distinguishable through the dendrogram analysis. Since there is no *V. cholerae* and only one strain of *V. albensis* in the Bruker MBT Library, all *V. cholerae* were initially erroneously identified as *V. albensis*. In this study, we found that due to the small number of strains of *V. furnissii* and *V. fluvialis*, the MSP of one strain of *V. furnissii* we generated was clustered into the group of *V. fluvialis*. Similarly, the pairs *V. ordalii* and *V. gazogenes* plus *V. mytili* and *V. harveyi* also were clustered into each other’s species group. However, this does not necessarily mean that they are difficult to distinguish and require a large number of strain MSPs to support further inferences. The number of strains in the Bruker MBT Library is small, and most of them come from countries outside of China. There may be some differences in Chinese strains such that they do not adequately reflect the diversity of the *Vibrio* species spectra. Certainly, our findings suggest that including only one MSP in the database is not sufficient for species identification, perhaps partly due to high heterogeneity among different regions.

Expanding the database could improve the identification accuracy of indistinguishable species. Although the Bruker MBT Library is the most widely used database, it lacks coverage of environmental bacterial isolates, particularly marine bacteria (Emami et al., 2012). Therefore, the use of this database for environmental surveys is limited. The number of strains of potentially pathogenic *Vibrio* species in the latest database of the Bruker MBT Library is small. This also explains why *V. alginolyticus* was misidentified as *V. mytili*, *V. parahaemolyticus* or *V. harveyi* when identified using the Bruker MBT Library. These species are nearly indistinguishable from each other by 16S rRNA gene analysis, requiring the analysis of multiple housekeeping genes for accurate species identification (Thompson et al., 2005, 2007). The result of 16S rRNA gene analysis showed that the *V. alginolyticus* strains exhibited 56–80% similarity with *V. parahaemolyticus* strains. The MSPs of *V. parahaemolyticus* exhibited 40–45% similarity to those of *V. mimicus* (Hazen et al., 2009). It is worth mentioning that the dendrogram analysis of the *Vibrio* species in the Bruker MBT Library and PVBase similarly indicated a close genetic relationship among *V. alginolyticus*, *V. mytili*, *V. parahaemolyticus* and *V. harveyi* that further explains why some strains of *V. alginolyticus* were misidentified initially. However, *V. alginolyticus* could be accurately identified after expanding the database. Furthermore, after adding MSPs of *V. cholerae* to the database, all *V. cholerae* were accurately identified. It is clear that augmenting the database makes it possible to discern otherwise indistinguishable species.

The *Vibrio* strains in this study came from different regions in China and included environmental and clinical strains. We analyzed the characteristics of *Vibrio* strains from different regions or environmental and clinical strains in order to explore the influence of the above factors on the MSP characteristics of *Vibrio* species. However, we did not find statistically significant patterns. Although clinical strains are mainly derived from hospital patients, patients may be infected from various regions, so this may have confounded the analysis. Previous studies showed that the characteristics of *Vibrio* species from different regions may differ (Hsueh et al., 2004; Mishra et al., 2011) and that geographic diversity could lead to divergences in MALDI-TOF MS profiles (Hazen et al., 2009; Li et al., 2017, 2018). For example, the MALDI-TOF MS spectra of the strains of *V. parahaemolyticus* isolated from Japan, exhibit more similarities than spectra of the strains isolated from the United States (Hazen et al., 2009). Differences in the spectra of some of the *V. parahaemolyticus* strains may reflect strain adaptation to a particular geographic location. However, a *V. parahaemolyticus* environmental strain isolated from Florida exhibited similarities to the environmental strains isolated from North Carolina (Hazen et al., 2009). This may also explain why we did not find statistical differences in the characteristics of MSPs between different regions or between environmental and clinical strains of the same *Vibrio* species. Although our strains come from a wide range of regions in China, they all belong to the coastal areas connecting Oceania, and the extensive exchanges between regions may eliminate differences to a certain extent. Moreover, it may be that all strains are from China, and that domestic differences are not enough to distinguish them (Morita et al., 2020). In the future, we hope to obtain more strains from abroad for analysis and comparison with strains from China, to consider regional differences at a larger scale. Finally, we would like to open the use of the database, and will share the PVBase database with other scholars to maximize resource sharing and improve the identification efficiency of potentially pathogenic *Vibrio* species.

## Data Availability Statement

The raw data supporting the conclusions of this article will be made available by the authors, without undue reservation. All main spectra of PVBase are available as btmsp file as a supplement.

## Author Contributions

JLW, BZ, and WX: conceptualization and writing—review and editing. BZ, TL, and WX: methodology. LK and JL: software. TL, LK, and JX: validation. TL, JX, and WX: formal analysis. JW, SG, and JX: investigation. YL and BY: resources and data curation. TL, JX, and LK: writing—original draft preparation and visualization. YY and YL: supervision. BZ, JLW, and WX: project administration. BZ, JLW, and WX: funding acquisition. All authors have read and agreed to the published version of the manuscript.

## Conflict of Interest

The authors declare that the research was conducted in the absence of any commercial or financial relationships that could be construed as a potential conflict of interest.

## Publisher’s Note

All claims expressed in this article are solely those of the authors and do not necessarily represent those of their affiliated organizations, or those of the publisher, the editors and the reviewers. Any product that may be evaluated in this article, or claim that may be made by its manufacturer, is not guaranteed or endorsed by the publisher.

## Footnotes

1 http://www.bacterio.net/vibrio.html; accessed on 15 December 2021.
