# Supplementary material for: PVBase: A MALDI-TOF MS Database for Fast Identification and Characterization of Potentially Pathogenic Vibrio Species From Multiple Regions of China
Source: Front Microbiol. 2022 May 17;13:872825. doi: 10.3389/fmicb.2022.872825 (PMC9152771; doi:10.3389/fmicb.2022.872825)
Supplement: Supplementary file 1 [file Table_1.DOCX]

Supplementary Material


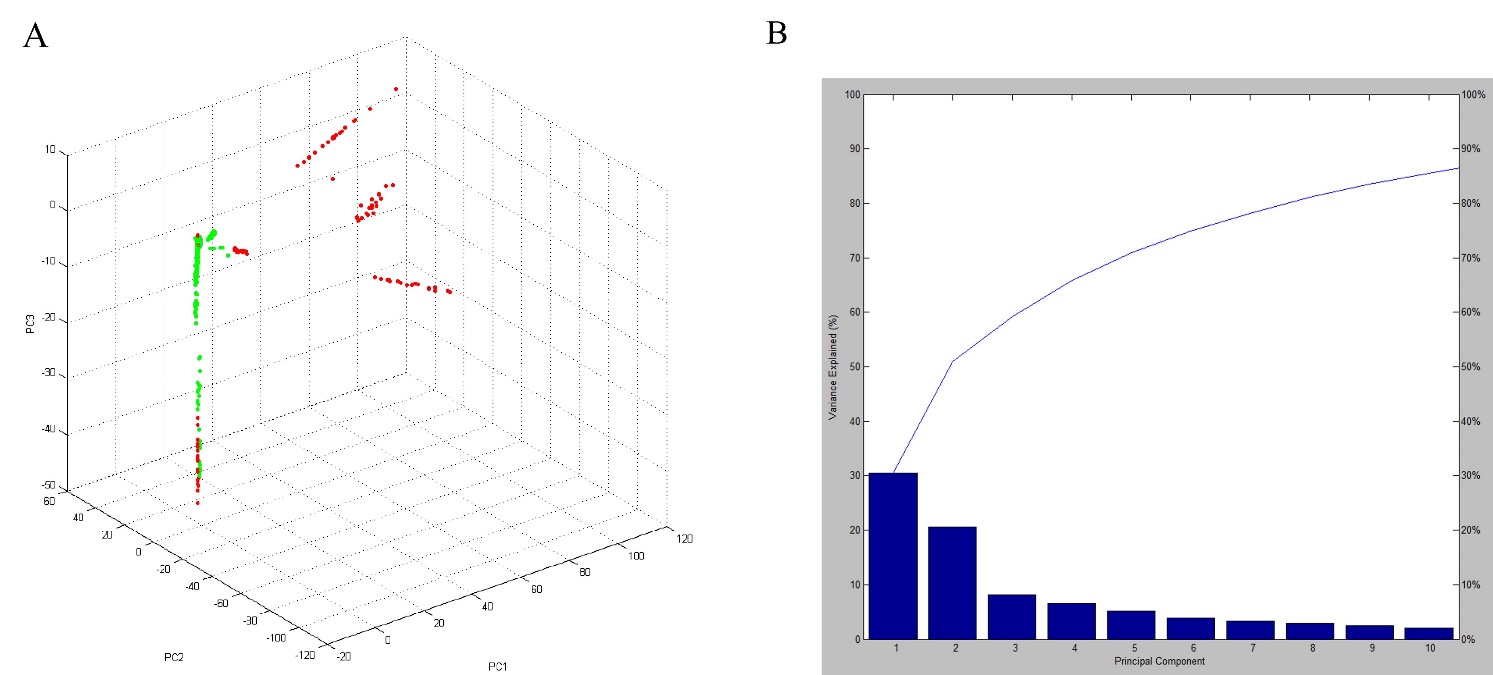


**Supplementary Figure 1.** PCA analysis of *V. parahaemolyticus* in northern and southern China. **(A)** The result of PCA analysis. The red points represent *V. parahaemolyticus* in northern China. The green points represent *V. parahaemolyticus* in southern China. **(B)** The variance value of each principal component (PC).
